# Quantitative Analysis of the Protein Methylome Reveals PARP1 Methylation is involved in DNA Damage Response

**DOI:** 10.3389/fmolb.2022.878646

**Published:** 2022-06-29

**Authors:** Xinzhu Wang, Shaojie Mi, Mingxin Zhao, Chen Lu, Chenxi Jia, Yali Chen

**Affiliations:** ^1^ Jiangsu Key Laboratory of Marine Pharmaceutical Compound Screening, Jiangsu Ocean University, Lianyungang, China; ^2^ State Key Laboratory of Proteomics, National Center for Protein Sciences—Beijing, Beijing Proteome Research Center, Beijing Institute of Lifeomics, Beijing, China; ^3^ Co-Innovation Center of Jiangsu Marine Bio-industry Technology, Jiangsu Ocean University, Lianyungang, China; ^4^ Key Laboratory of Industrial Fermentation Microbiology, Tianjin Industrial Microbiology Key Lab, Ministry of Education, College of Biotechnology, Tianjin University of Science and Technology, Tianjin, China; ^5^ Jiangsu Marine Resources Development Research Institute, Jiangsu Ocean University, Lianyungang, China

**Keywords:** protein methylation, DNA damage response, HILIC affinity enrichment, PARP1, ionizing radiation

## Abstract

Protein methylation plays important roles in DNA damage response. To date, proteome-wide profiling of protein methylation upon DNA damage has been not reported yet. In this study, using HILIC affinity enrichment combined with MS analysis, we conducted a quantitative analysis of the methylated proteins in HEK293T cells in response to IR treatment. In total, 235 distinct methylation sites responding to IR treatment were identified, and 38% of them were previously unknown. Multiple RNA-binding proteins were differentially methylated upon DNA damage stress. Furthermore, we identified 14 novel methylation sites in DNA damage response-related proteins. Moreover, we validated the function of PARP1 K23 methylation in repairing IR-induced DNA lesions. K23 methylation deficiency sensitizes cancer cells to radiation and HU-induced replication stress. In addition, PARP1 K23 methylation participates in the resolution of stalled replication forks by regulating PARP1 binding to damaged forks. Taken together, this study generates a data resource for global protein methylation in response to IR-induced DNA damage and reveals a critical role of PARP1 K23 methylation in DNA repair.

## Introduction

Different genotoxic factors can produce multiple types of DNA damage (Ciccia and Elledge, 2010; Chatterjee and Walker, 2017). Among them, the most hazardous one is double-strand breaks (DSBs). If improperly removed or repaired, these DNA lesions will be cytotoxic and threaten the integrity of the genome. Cells have evolved a complex and effective DNA damage response (DDR) system to sense and repair DNA damage (Jackson and Bartek, 2009). Defective DDR machinery leads to genomic instability, which in turn drives tumorigenesis. For example, the BRCA1 gene mutations were found in approximately 50% of familial breast cancers. Exploring the mechanisms in the DDR system can provide potent treatment strategies for individual tumors. A well-known example is that PARP inhibitors (PARPi) have been successfully used in cancers with BRCA1 and BRCA2 mutations. Therefore, studying the DDR pathway has important implications in cancer pathogenesis and cancer therapy.

Posttranslational modifications (PTMs) are well-known modulators of DNA damage signaling (Polo and Jackson, 2011; Dantuma and van Attikum, 2016). Many reports have revealed that both DDR proteins and chromatin components can be modified via phosphorylation, acetylation, ubiquitination, methylation, etc. Such modifications regulate the DDR pathways in a strictly orderly way. Protein methylation is one of the most ubiquitous PTMs in the DDR system (Chen and Zhu, 2016; Urulangodi and Mohanty, 2020). Protein methylation commonly occurs at arginine (Arg) and lysine (Lys) in six forms, including mono-methylated, asymmetric di-methylated and symmetric di-methylated Arg and mono-, di-, and tri-methylated Lys. Protein arginine methylation is catalyzed by a family of protein arginine methyltransferases (PRMTs). Protein lysine methylation is catalyzed and regulated by lysine methyltransferases (KMTs) and lysine demethylases (KDMs) (Murn and Shi, 2017). In the past, protein methylation involved in the DDR was mainly observed on histones such as H3K4, H3K27, H3K36, H3K79, H4K20, and H2AXK134, which regulates the recruitment of different DDR factors to DNA damage sites (Chen and Zhu, 2016). In recent decades, nonhistone substrates, such as 53BP1, MRE11, BRCA1, PARP1, RUVBL1, TDP1, hnRNPUL1, and TOP3B, have been found to be methylated and identified as functional in the DDR process (Chen and Zhu, 2016). In addition, several methyltransferases (especially PRMT1 and PRMT5) were also characterized as possible DDR factors (Auclair and Richard, 2013). The PRMT1 knockout MEFs revealed spontaneous DNA damage and chromosomal instability, suggesting that PRMT1 is a player in the DDR pathway. Some known DDR proteins (53BP1, BRCA1, MRE11, POLB, etc.) have been identified as targets of PRMT1 and PRMT5 (Auclair and Richard, 2013). For example, the R1398, R1400 and R1401 sites of 53BP1 could be methylated by PRMT1, which promoted its binding to DNA and facilitated the NHEJ pathway (Boisvert et al., 2005). And the di-methylation by PRMT5 increases the stability of 53BP1 and promotes NHEJ repair (Hwang et al., 2020). Although many studies have suggested that protein methylation plays an important role in the DDR, there is still a lack of a thorough profiling of whole-cell methylation changes during the DNA damage response and repair.

The global profiling of methylated endogenous peptides in cells is challenging due to the low abundance and low stoichiometry of many methylated molecules. Therefore, enrichment of methylated peptides is often needed for methylation identification. Various techniques have been developed, such as antibody-based immunoaffinity purification (Guo et al., 2014), strong-cation exchange chromatography (SCX) (Wang et al., 2016), and hydrophilic interaction chromatography (HILIC) (Uhlmann et al., 2012). Among them, HILIC is simple and easy to perform and is not limited to peptide sequences or methylation types (Uhlmann et al., 2012). At the same time, HILIC is suitable for the combination of mass spectrometry (MS) or evaporative light scattering detection (ELSD). Currently, HILIC has become an important method for large-scale enrichment and identification of intracellular methylated peptides (Yan et al., 2019).

Poly (ADP-ribose) polymerase-1 (PARP1) is one abundant nuclear protein and catalyzes the transfer of the ADP-ribose unit from NAD + to target proteins. Through the poly-ADP-ribosylation of target proteins such as histones, PARP1 functions in DNA repair, chromatin modification, and transcriptional regulation. PARP1 plays as a nick-sensor and contributes to organizing the DNA repair machinery, in the context of both DNA single-strand breaks (SSBs) and DSBs (Pascal, 2018). The DNA damage-driven activation of PARP1 recruits enzymes, including XRCC1, DNA ligase III, DNA polymerase β, and MRE11, to DNA damage sites (Prasad et al., 2001; El-Khamisy et al., 2003; Leppard et al., 2003; Bryant et al., 2009). Inhibition of PARP1 has been proven to enhance the cytotoxicity of DNA-damaging agents in cancer treatments. Mammalian PARP1 is a 116-kDa protein comprising an N-terminal DNA-binding domain, a central auto-modification domain, and a C-terminal catalytic domain. The auto-modification domain contains several glutamate, aspartate and lysine residues necessary for its auto-ADP-ribosylation. It has been reported that PARP1 can be modified through multiple posttranslational modifications, including phosphorylation, acetylation, sumoylation, ubiquitination and ADP-ribosylation (Piao et al., 2018). Regarding methylation, several recent reports have mentioned that PARP1 could be methylated at its K508 and K528 sites by SET7 and SMYD2, respectively (Kassner et al., 2013; Piao et al., 2014). These methylations positively regulated the poly-ADP-ribosylation activity of PARP1 and assured efficient PAR formation upon oxidative stress. Meanwhile, whether there are still other mechanisms by which PARP1 activity is controlled remains to be elucidated.

In this study, using HILIC affinity enrichment combined with MS analysis, we systematically analyzed the changes in methylated proteins and modification sites in HEK293T cells before and after IR treatment. We determined the potential connections of these modified proteins with the IR-induced DNA damage response. PARP1 is a well-known DNA damage protein, and the function of a novel methylation of PARP1 in DDR pathways was also investigated. We found that the K23 methylation of PARP1 was important for the repair of IR-induced DNA lesions. K23 methylation deficiency sensitizes cancer cells to radiation and replication stress. In addition, PARP1 K23 methylation promotes DNA repair mainly by regulating the resolution of stalled replication forks. These results suggest that the K23 methylation of PARP1 is a novel factor in the DNA damage response. Because approximately 38% of the identified methylated peptides were previously unknown, we think they might provide meaningful clues for further investigations.

## Materials and Methods

### Cell Culture

HEK-293T, U2OS, MDA-MB-231 cells were cultured in Dulbecco’s modified Eagle medium (DMEM; high glucose, Gibco) supplemented with 10% fetal bovine serum (FBS) and 1% penicillin/streptomycin at 37°C with 5% CO_2_.

### Antibodies and Constructs

Antibodies against poly-PAR (4336-BPC-100, Trevigen, IF: 1:200), PARP1 (9532S, Cell Signaling Technology, WB: 1:1000), BRCA1 (D-9, Santa Cruz, IF: 1:100), HA (H9658, Sigma, WB 1:2000), γH2AX (05-636, Merck Millipore, IF: 1:1000), 53BP1 (NB100-304, Novus, IF: 1:100) and BrdU (ab6326, Abcam, IF: 1:200) were used. Full-length PARP1 was subcloned into the HA-tagged vector (pCMV-HA), the GFP-tagged vector (pFUGW) or the lentiviral vector (pHAGE-EF-Puro-DEST). The shPARP1 plasmid was constructed as described. The K23A and K418A mutant PARP1 plasmids were generated by site-directed mutagenesis and confirmed by sequencing. The shPARP1 stable cell lines and the reconstituted wild-type (WT), K23A and K418A mutant PARP1 stable cell lines were constructed according to http://www.addgene.org/protocols/plko/#C, and lentiviral packaging vectors pMD2.0G and pSPAX from NovoPro Bioscience were used.

### Sample Preparation

HEK-293T cells were irradiated (10 Gy, with a PXi X-RAD 160 X-ray Irradiator) at a dose rate of 1.907 Gy/min) and harvested 2 h later. Three biological replicates were included for the comparative proteomics in cells with and without irradiation. Cells were washed and harvested with 1× PBS buffer (Gibco) and lysed with lysis buffer [20 mM HEPES, pH 8.0; 9 M urea; 1 mM sodium orthovanadate; 2.5 mM sodium pyrophosphate; 1 mM β-Glycerophosphate, and protease inhibitor cocktail (Merck)]. The lysate was sonicated and then centrifuged at 15000 rpm at room temperature for 15 min. The supernatant was collected and transferred into a new tube, and the concentration was determined using a BCA Protein Assay kit (TIANGEN). Proteins were digested using the FASP protocol: 1 mg protein was reduced with 10 mM DTT at 55°C for 1 h and alkylated with 30 mM iodoacetamide (IAA) at room temperature for 30 min under dark conditions. The solution was transferred to a 10-kDa-MWCO filter (Millipore) and washed three times with 20 mM HEPES (pH 8.0). Add 0.4 mL 20 mM HEPES (pH 8.0) and trypsin (the ratio of enzyme to protein is 1:50 w/w) to the ultrafiltration tube and incubate the enzyme digestion overnight at 37°C.

### Peptide Enrichment and LC–MS/MS Analysis

HILIC beads were activated with 0.5% TFA (trifluoroacetic acid) for 5 min and 0.5% FA (fluoroacetic acid) for 10 min, washed with 0.5% FA three times, equilibrated with 80% ACN and 5% FA for 5 min, and the supernatant was discarded. The digested peptides were dissolved in 100 μL of balance solution (80% ACN, 5% FA), and the above activated beads were added to fully bind for 10 min. The elution of peptides was performed using prepacked two-layer C18 membrane columns. The columns were first rinsed with balance solution 5 times and then eluted with 0.5% FA three times. The three eluted solutions were combined and evaporated to dryness.

The methylated peptide sample was resuspended in water with 0.1% FA at a concentration of 0.1 μg/μL and analyzed on an EASY-nLC 1200 nano-HPLC system (Thermo Fisher Scientific) coupled to a Q Exactive HF Orbitrap mass spectrometer (Thermo Fisher Scientific). Five microliters of samples were uploaded on a trap column (3 mm C18 particles, 2 cm × 100 µm ID) and then separated on a 30-cm analytical column (1.9 µm C18 particles, 30 cm × 150 µm ID) at a 600-µL/min flow rate for 78 min. An aqueous solution with 0.1% FA and 80% ACN with 0.1% FA was utilized as solvents A and B, respectively. The electrospray voltage was 2.0 kV. The mass spectrometer was operated in data-dependent acquisition mode with a resolution of 120,000 in full scan mode. The maximum injection time was 80 ms for full scans and 100 ms for MS/MS scans, and dynamic exclusion of previously acquired precursor ions was enabled at 25 s. A survey scan was acquired after accumulating 5e5 ions in Orbitrap for m/z 300–1400; the top 30 most intense ions in each scan were automatically selected for HCD fragmentation with a normalized collision energy of 32% and measured in an Orbitrap analyzer operating at a resolution of 15,000.

### Database Search

The mass spectrometry raw data files were analyzed using PEAKS Studio 8.5 (Bioinformatics Solutions Inc., Canada). All three technical replicates were searched against a human protein database downloaded (9 Mar 2017) from UniProt encompassing 20166 entries. A *de novo* process was performed before the database search. The mass tolerance was set to 10 ppm for precursor ions and 0.02 Da for fragment ions. Mono-methylation (K/R), di-methylation (K/R), trimethylation (K), and oxidation (M) were set as variable modifications, and carbamidomethylation (C) was set as a fixed modification. Trypsin cleavage was selected, and up to 3 variable posttranslational modifications were allowed for each peptide. The discovered peptides were filtered by a false discovery rate (FDR) of 1% and an A-score of 20. Label-free relative quantitation parameters within the PEAKS software were used to generate normalized protein intensities.

### Protein Functional Annotation

Online WebLogo (Crooks et al., 2004) was used to analyze the sequence characteristics of methylated peptides. The DAVID (Database for Annotation, Visualization, and Integrated Discovery) database was utilized to conduct GO analysis (Huang et al., 2009). The Search Tool for Recurring Instances of Neighbouring Genes (STRING) database was used to annotate interacting protein networks (Szklarczyk et al., 2019; Szklarczyk et al., 2021), and the Ingenuity Pathway Analysis (IPA) database was used for signal pathway analysis (Kramer et al., 2014).

### Western Blot Analysis

Total cell lysates were prepared by lysing cells in NETN buffer (10 mM Tris-HCl, pH 8.0, 100 mM NaCl, 1 mM EDTA, and 0.5% NP-40) with protease inhibitors (Merck) on ice for 30 min. The lysates were cleared by centrifugation at 16000 rpm for 10 min. The samples for western blotting were prepared by mixing the supernatant with equal volumes of 2× sample loading buffer (100 mM Tris–HCl pH 6.8, 100 mM DTT, 4% SDS, 0.2% bromophenol blue, 20% glycerol). Equal amounts of total protein were separated by SDS–polyacrylamide gel electrophoresis and then transferred to a nitrocellulose membrane (PALL) and immunoblotted with antibodies. The membrane was further washed with TBST and developed using ECL reagents (CWBIO).

### Immunofluorescence Staining

U2OS cells were seeded on poly-lysine-coated coverslips or in 35 mm petri dishes with light-transmitting glass at the bottom. After IR treatment (10 Gy, then recovered for the indicated time course), the cells were washed 3 times with 1× PBS, fixed in 4% paraformaldehyde for 15 min and permeabilized in 0.5% Triton X-100 solution for 5 min at room temperature. Cells were then blocked with 5% bovine serum albumin solution for 1 h and incubated with primary antibody overnight at 4°C. The coverslips were washed three times with 1× PBS, and secondary antibody was applied for 30 min at 37°C. The nucleus was counterstained with DAPI at room temperature for 1 min and then mounted with antifade solution. All the samples were visualized by a Nikon A1R fluorescence microscope and Nikon NIS software. Quantitative data were analyzed with ImageJ 1.53C software.

### Colony Formation Assay

U2OS cells were seeded at a low density and treated with IR and HU at the indicated doses. The cells were washed with 1× PBS twice and then cultured at 37°C for 14 days. Cells were fixed with 1 mL paraformaldehyde for 15 min and then washed twice with 1× PBS. Colonies were stained with 0.25% (w/v) crystal violet for 30 min and washed gently with water. Colonies were counted, and statistical data were analyzed by t test analysis. Data are presented as the mean ± SEM (standard error mean) of three independent experiments.

### CldU Coimmunoprecipitation

Cells were treated with 2 mM HU for 3 h and 100 μM CldU for 30 min. Cells were fixed and cross-linked with 1% formaldehyde and then treated with 0.125 M glycine for 15 min at room temperature. Cells were harvested by scraping in cold 1× PBS. Cytoplasmic proteins were removed by incubation in hypotonic buffer (10 mM HEPES pH 7, 50 mM NaCl, 0.3 M sucrose, 0.5% Triton X-100, protease inhibitors (Merck)) for 10 min on ice and centrifugation at 4000 rpm for 5 min. The nuclear soluble fraction was removed by nuclear buffer [10 mM HEPES pH 7, 200 mM NaCl, 1 mM EDTA, 0.5% NP-40, protease inhibitors (Merck)] for 10 min on ice and centrifugation at 13000 rpm for 5 min. Cells were resuspended in lysis buffer [10 mM HEPES pH 7, 500 mM NaCl, 1 mM EDTA, 1% NP-40, protease inhibitors (Merck)] and sonicated at 14% power for 1 min with a probe sonicator (Scientz-IID, China). The cell lysate was cleared by centrifugation at 16000 rpm for 20 min. The protein concentration of the lysates was determined using a BCA Protein Assay kit (TIANGEN). A total of 500 μg of protein was used for the IP reaction with 2 μg of anti-BrdU antibody [BU1/75(ICR1), Abcam] and 20 μL of protein G agarose (Sigma). The reaction was washed twice with nuclear buffer and washing buffer [10 mM HEPES, 0.1 mM EDTA, protease inhibitors (Merck)] and incubated in 2× sample loading buffer (100 mM Tris–HCl pH 6.8, 100 mM DTT, 4% SDS, 0.2% bromophenol blue, 20% glycerol) for 30 min at 90°C and used for western blotting as described above.

## Results

### Proteomic Analysis of the Methylated Peptides and Proteins Following IR

**FIGURE 1:**
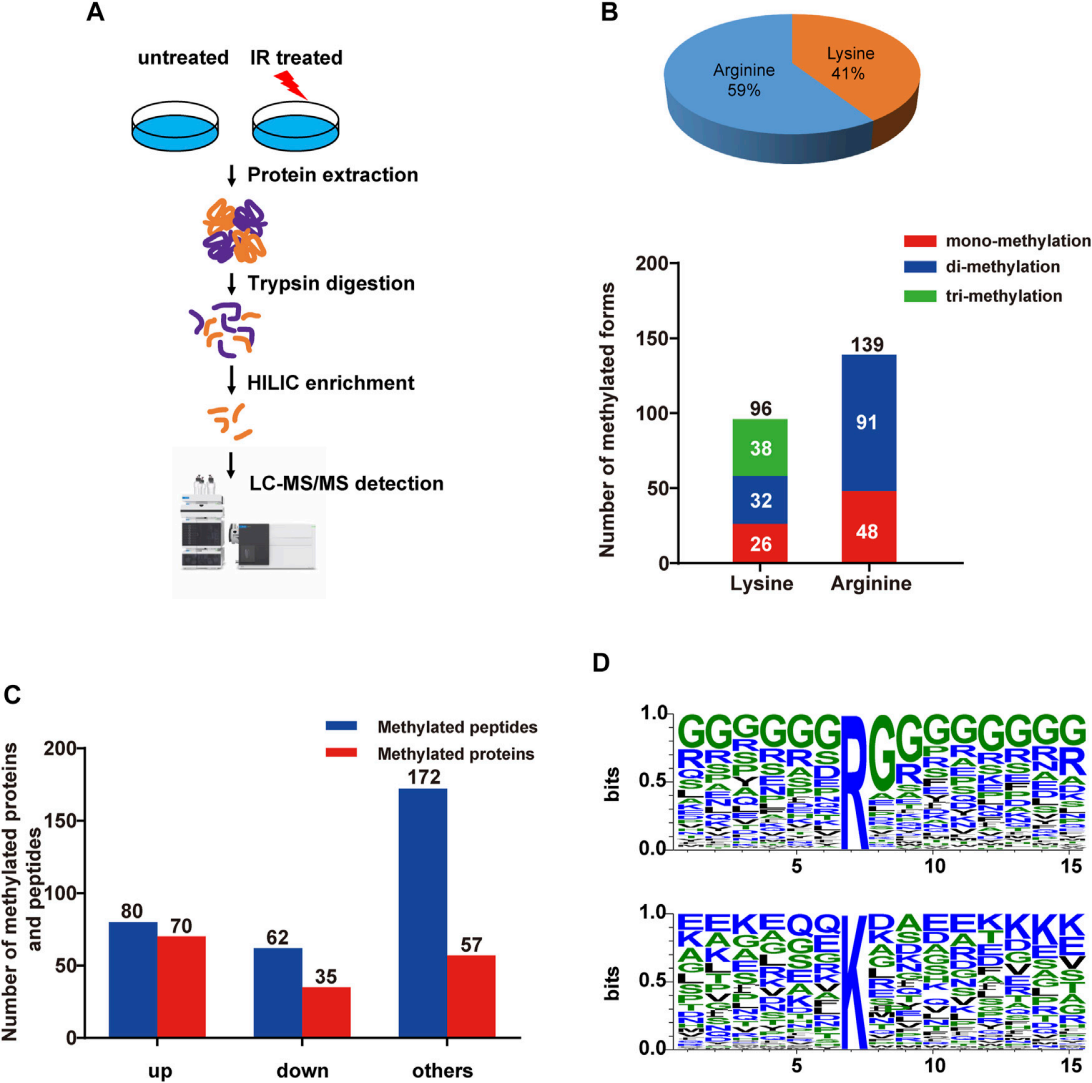
Quantitative overview of methylated peptides and proteins in HEK293T cells upon IR treatment. **(A)** The strategy for global analysis of protein methylation in response to IR treatment. HEK293T cells were treated with IR, and then proteins from cell lysates were digested with trypsin. The HILIC tip enrichment was processed. Then, large-scale identification of protein methylation was carried out by LC−MS/MS followed by data analysis and bioinformatics analysis. **(B)** Number of methylated proteins and peptides quantified in HEK293T cells in response to IR treatment. Upper panel: ratio of lysine and arginine methylation sites; lower panel: numbers of different methylation forms. **(C)** Bar plots showing the identified up- or down-represented (log2-fold change>1 and <−1) methylated peptides and proteins upon IR-induced DNA damage. ‘up’, methylated peptides and proteins whose intensities were increased at least 2-fold; ‘down’, methylated peptides and proteins whose intensities were increased at least 2-fold; ‘others’, methylated peptides and proteins whose intensities were changed less than 2-fold. **(D)** Sequence logo of identified arginine (R) and lysine (K) methylated sites within −6 to +8 amino acids, generated with WebLogo. The overall height of each stack indicates the sequence conservation at that position (measured in bits), whereas the height of symbols within the stack reflects the relative frequency of the corresponding amino or nucleic acid at that position.

Ionizing radiation (IR) is a common inducer of DNA damage, including double-strand breaks, and threatens genome stability. However, the global characterization of methylation modifications during the DNA damage response to IR treatment has not been reported before. In our study, we performed HILIC enrichment combined with MS identification of methylated proteins from HEK293T cells with or without IR treatment (5 Gy) (Figure 1A). By applying strict quality control (FDR at the peptide and sites level to be <1%, A-score to be > 20), we obtained 314 differentially methylated peptides and determined 235 distinct methylation sites, combining the results from three technical replicates (referring to Datasheet 1, Sheet 1). Among them, we identified 96 lysine methylation sites (including 26 mono-methylation sites, 32 di-methylation sites and 38 trimethylation sites) and 139 arginine methylation sites (including 48 mono-methylation sites and 91 di-methylation sites) (Figure 1B, referring to Datasheet 1, Sheet 1). These peptides and sites were mapped to 162 human proteins. Among them, 80 peptides with 80 sites showed at least 2-fold increase of abundance in IR-treated group than untreated group, while 62 peptides with 52 methylation sites showed at least 2-fold decrease of abundance in IR-treated group (Figure 1C, referring to Datasheet 1, Sheet 2). Compared to the PhosphoSitePlus^®^ database (Hornbeck et al., 2015), more than 38% of the changed methylation sites were novel modifications (Datasheet 1, Sheet 3). At the same time, 34% of the identified methylated proteins have been reported to be previously involved in the DNA repair process, such as histone H3, FUS, SFPQ, and DHX9 (Datasheet 1, Sheet 4). For example, FUS is a well-known DNA/RNA-binding protein that plays a role in various cellular processes, such as transcription regulation, RNA splicing, RNA transport, DNA repair and damage response. Recent research demonstrated that FUS-dependent liquid–liquid phase separation (LLPS) is necessary for the initiation of the DDR (Levone et al., 2021). Here, we identified di-methylated R473, R476, and R481 sites within the FUS C-terminal RGG-rich domain, which has been reported to contribute to the phase separation of FUS (Hofweber et al., 2018), indicating that such methylation sites might take part in the regulation of the DDR. As a positive control, three known DDR-related histone methylation sites were determined in this work, including H3K27 mono-, di- and tri-methylation, K36 mono- and tri-methylation and K79 mono-, di-methylation (Kim et al., 2019), further demonstrating the efficacy of our screen.

To determine whether there were any potential motif sequences surrounding the identified methylation sites, we analyzed the frequencies of amino acids in methylation site-adjacent residues (from −6 to +8) (Figure 1D). Although there were no definite motifs, the basic amino acid lysine (K) and the acidic glutamic acid (E) showed somewhat overrepresentation around the methylated lysine. Lysine was enriched, especially at the upstream −4 positions and downstream +6 to +8 of the methylated lysine. Glutamic acid was more enriched at the upstream positions −1 and −5 and at the downstream +3, +4, and +8 positions. Additionally, glutamine was overrepresented at positions −1 and −2. We observed a highly abundant glycine around the arginine methylation site (almost from upstream −6 to downstream +8), which acts as the RGG motif for specific PRMTs. However, lysine methyltransferases might have no distinct target motifs.

### Functional Annotation of Identified Methylated Proteins Upon IR Treatment

**FIGURE 2:**
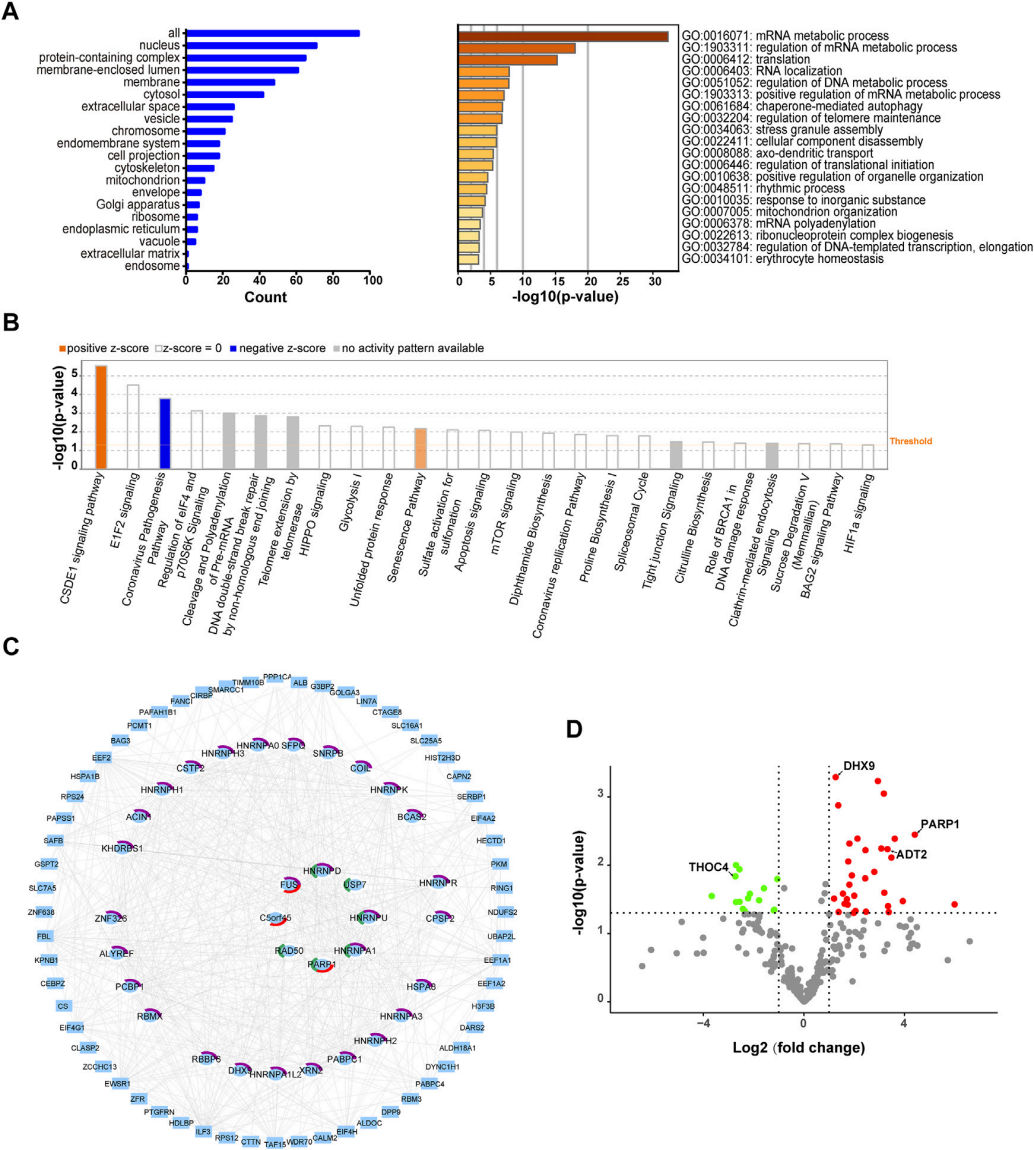
Subcellular localization and functional annotation of identified methylated proteins upon IR treatment. **(A)** Left: The subcellular distribution of methylated proteins in HEK293T cells upon IR treatment. Right: Functional classification of identified proteins based on GO analysis. **(B)** Top enriched canonical pathways based on IPA analysis. The top 25 significant canonical pathways are shown ordered by significance (*p*-value), calculated in IPA by right-tailed Fischer’s exact t-test. A positive z-score (orange) indicates that a pathway activity is increased, while a negative z-score (blue) indicates a pathway activity is decreased. And no activity pattern available (grey) was included here. **(C)** Protein–protein interaction (PPI) network of proteins with altered methylation upon IR treatment. Methylated proteins derived from the data (log2-fold change >1 and <−1) were analyzed with STRING. The proteins involved in GO:1905168 (positive regulation of double-strand break repair via homologous recombination) and in GO:0032204 (regulation of telomere maintenance) are highlighted in red and green, respectively. The proteins counted in GO:0006397 (mRNA processing) are labelled in purple. **(D)** Volcano plot showing the most significantly regulated methylation peptides upon IR treatment. The red circle represents *p* < 0.05 and log2-fold change >1; the green circle represents *p* < 0.05 and log2-fold change < −1. The representative differentially methylated proteins in the DDR pathway are listed.

To explore the impact of differentially methylated proteins on cell physiological processes and discover internal relationships between them, we performed GO analysis on the 96 identified methylated proteins that were considered significantly changed (log2-fold change>1 and <−1). As shown in Figure 2A, most (approximately 76%) methylated proteins resided in the nucleus, while 69% occurred in protein-containing complexes. We further checked the methylated proteins based on their molecular functions and biological processes. Proteins involved in mRNA and DNA metabolic processes were the most abundant proteins upon IR treatment, and a large portion of them were spliceosome components or possessed RNA-binding activity, indicating that many RNA-binding proteins might be differentially methylated in response to DNA damage stress. In addition, some proteins involved in the cell stress response process were also enriched in our study, including the regulation of telomere maintenance and stress granule assembly, also suggesting their roles in the DNA damage response (Figure 2A) (Slijepcevic, 2008; Pothof et al., 2009).

The Ingenuity® Pathway Analysis (IPA) is a powerful tool to identify significant networks, and canonical pathways associated with the differentially expressed genes (Li et al., 2015). For the above 96 methylated proteins that respond to IR-induced DNA damage (log2-fold change>1 and <−1), we performed an IPA analysis to identify the canonical pathways involved. A graphical summary of the top 25 canonical pathways was provided in Figure 2B. We found that CSDE1 signaling and senescence were two canonical pathways with positive Z score (Kramer et al., 2014), indicating the activation of these pathways in IR-treated cells. CSDE1 signaling pathway was the top canonical pathway (-log (*p*-value) = 5.54, Ratio = 0.0893). The identified components of this pathway (CTTN, FUS, HNRNPD, PABPC1, PABPC4) were implicated in mRNA turnover and efficient formation of stress granules (Lindquist and Mertens, 2018; Youn et al., 2018). Such stress granules have been illustrated as part of cellular stress response. Likewise, the identified factors of the senescence pathway (-log (*p*-value) = 2.18, Ratio = 0.0168), including CAPN2, PARP1, PPP1CA, RAD50, RING1, have been demonstrated as stress response elements to DNA damage (von Zglinicki et al., 2005; d'Adda di Fagagna, 2008). The activation of these two pathways suggested that the methylations of their components could be necessary for the DDR, probably through shaping mRNA processing or regulating senescence. Not surprisingly, two damage-related pathways were also identified among the top 10: DNA double-strand break repair by nonhomologous end joining (-log (*p*-value) = 2.86, Ratio = 0.143) and telomere extension by telomerase (-log (*p*-value) = 2.8, Ratio = 0.133). Several components of these pathways, such as HNRNPA1, RAD50 and PARP1, have been shown as DDR regulators (Carney et al., 1998; Audebert et al., 2004; Audebert et al., 2006; Couto et al., 2011; Sui et al., 2015). The methylations of them perhaps modulate DDR.

To delineate the associations among the above 96 proteins with at least two-fold changes in methylation signals upon IR treatment, we performed network analyses of protein–protein interactions (PPIs) using STRING and Cytoscape software, applying high confidence score of ≥0.7. The most enriched interaction network was a mRNA processing pathway (GO:0006397) covering 29 proteins, including heterogeneous nuclear ribonucleoprotein family (ROA1/HNRNPA1, HNRNPD, HNRNPH3, etc.) and RNA-binding proteins (CIRBP, DHX9, SFPQ, FUS, RBMX, etc.) (Figure 2C). Among the 92 nodes (proteins mapped in STRING) and 246 edges (the number of interactions), FUS, ROA1/HNRNPA1, HNRNPD, HNRNPH3, and RBMX were the nodes with most interactions. More than 4 sites of these proteins have been differentially methylated upon IR treatment (Supplementary Figure S1A), implying that an altered methylation profile is involved in DDR pathway. As expected, we also found significant changes in the methylation levels of proteins related to double-strand break repair (GO:1905168) and telomere maintenance (GO:0032204) in response to IR-induced DNA damage (Figure 2C). A network analysis was performed with up or down-regulated methylated proteins (log2-fold change>1 or < −1) to unravel different pathways affected by methylation or de-methylation. RNA binding proteins (RBPs) were the most enriched factors in both up and down-represented proteins. Meanwhile, the factors implicated in the GO biological processes: ‘Positive regulation of DNA metabolic process’ and ‘Regulation of telomere maintenance’ were only enriched in up-represented group upon IR treatment (Supplementary Figure S1B). And the components implicated in the GO biological processes ‘Stress granule assembly’ were mainly enriched in down-represented group (Supplementary Figure S1C). These findings indicated that methylation of proteins in different pathways regulated the DDR in different ways.

Next, to explore possible differences in pathways affected by different froms of methylation, we analyzed the network of the up-represented (log2-fold change>1) K- methylome and R-methylome respectively. Both the K- and R-methylomes were mainly enriched with RNA binding proteins. Meanwhile, the K- and R-methylomes were implicated in unique biological processes. For instance, a cohort of proteins from the pathway ‘mRNA Splicing - Major Pathway (HSA-72163)’ were largely enriched in the R-methylome (Supplementary Figure S1D). And the factors from the pathways, namely, ‘Eukaryotic Translation Elongation (HSA-156842)’, ‘Cellular responses to stress (HSA-2262752)’, and ‘HDR through MMEJ (alt-NHEJ) (HSA-5685939)’, were mainly enriched in the K-methylome (Supplementary Figure S1E). The results revealed that R-methylation and K-methylation might involve different pathways to modulate the DDR. The comprehensive analysis of the methylation network might provide clues for further investigation.

To understand the role of differentially methylated proteins in the cellular response to IR-induced DNA damage, we compared the magnitude of changes and *p* values among the methylated peptides. The volcano plot (Figure 2D, referring to Datasheet 1, Sheet 5) depicts the most significantly regulated proteins (log2-fold change >1 and < −1, *p* < 0.05). The proteins with the most significantly up-regulated methylation signals in IR-treated cells included RING1, PARP1, PABP4, EEF1A2, XRN2, EF2, ADT2, DHX9, etc. The proteins with significantly downregulated methylation signals included RA1L2/HNRNPA1L2, FBL, RBP56, SFPQ, THOC4/ALYREF, etc (Datasheet 1, Sheet 5). Here, we discovered some methylation sites, which have been annotated in PhosphoSitePlus^®^ but have not been associated with DNA repair, such as K55me2 in EF1A1, R129me2 in HNRPH3, R204me2, R50me2, R38me2 of THOC4/ALYREF, and R851me2 in XRN2. In addition, we identified 14 novel methylation sites among the DDR-related proteins. Among them, the significantly changed (log2-fold change >1 and < −1, *p* < 0.05) sites included K418me in PARP1, K52me3 in ADT2, K58me3 and R72me2 in MRNIP, R283me in PAPS1, K409me in BAG3, and R167me2 in SPF27/BCAS2. The other 7 sites (namely, K1139me3 in RAD50, K588me3 in UBP7, K869me2 in FANCI, R573me2 in WDR70, K1463me2 in HECD1, R55me2 in IF4G1 and R105me2 in RBM3) were not significantly changed (log2-fold change >1 and < −1, *p* > 0.05) upon IR treatment.

### Characterization of Mono-Methylation of PARP1 at K23 Upon IR Treatment

**FIGURE 3:**
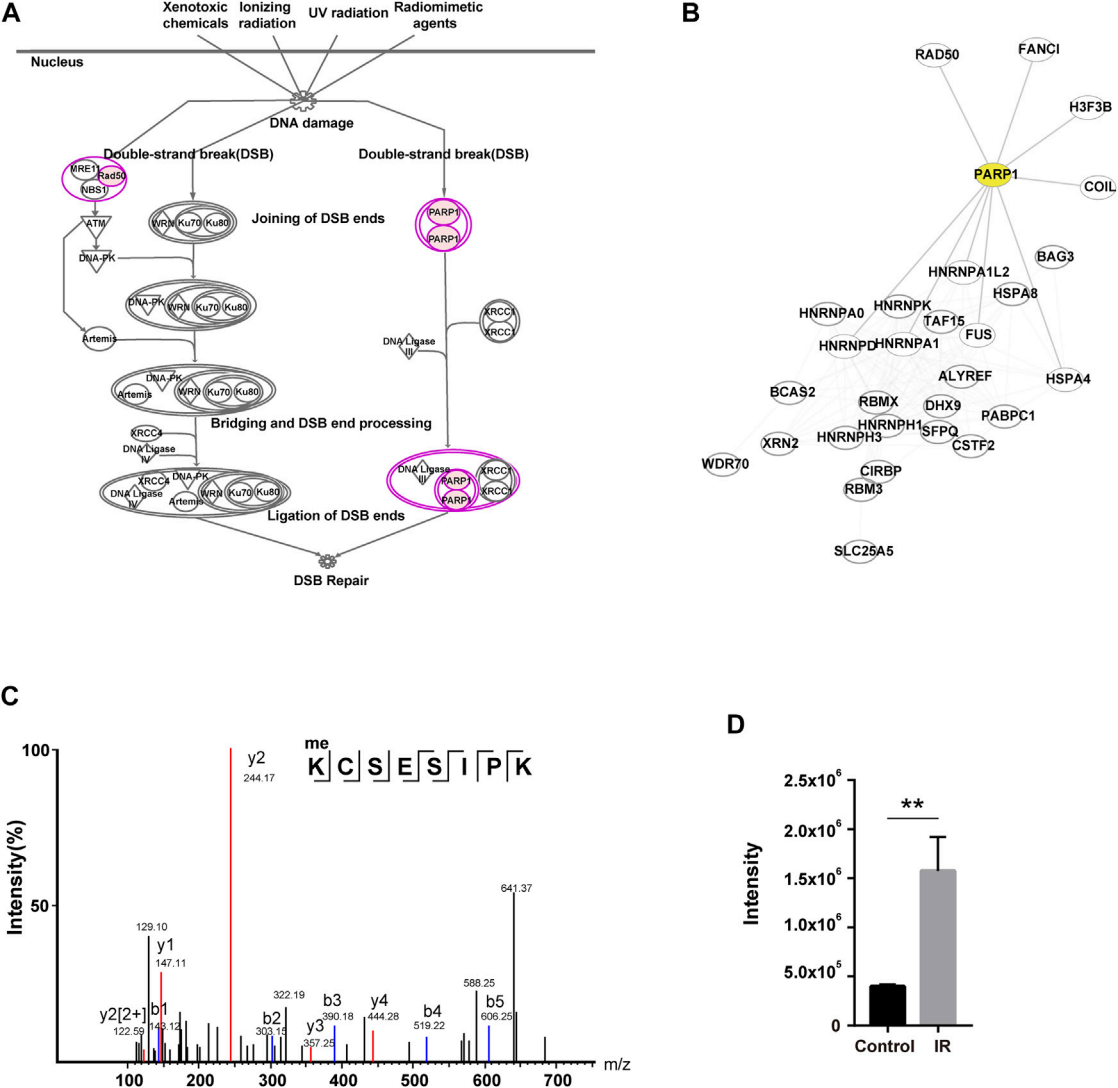
Characterization of a novel methylation of PARP1 upregulated in response to IR. **(A)** DNA damage pathway (DNA double-strand break repair by nonhomologous end joining) in IPA analysis. The identified PARP1 and RAD50 are shown in pink (log2-fold change>1). **(B)** PPI network of the DDR proteins in two DNA damage-related pathways identified with IPA. The proteins involved in pathways of DNA double-strand break repair by nonhomologous end joining and telomere extension by telomerase. PARP1 is highlighted in yellow. **(C)** The MS/MS fragmentation pattern of the methylated K23-containing peptide of PARP1. the B and Y ions for a given peptide represent the two halves formed by splitting the original peptide between various amino acids. For a given peptide sequence, the B ions are the product when the charge is retained on the N-Terminus (i.e. at the beginning of the sequence) and the Y ions the product when the charge is retained at the C-Terminus (i.e. at the end of the sequence). The identified B ions are shown in blue and Y ions are shown in red. **(D)** Quantification of the methylated K23-containing peptide of PARP1 in MS/MS with and without IR treatment. **p < 0.01.

As described above, there was one DNA damage-related pathway (DNA double-strand break repair by nonhomologous end joining) (Khanna and Jackson, 2001) among the top 10 pathways in IPA analysis. Two key players of this pathway, PARP1 and RAD50 (Carney et al., 1998; Audebert et al., 2004; Audebert et al., 2006; Wang et al., 2006; Couto et al., 2011), were methylated to a higher degree (log2-fold change>1) upon IR treatment (Figure 3A). The network analysis of the DDR proteins identified in the global methylome (Datasheet 1, Sheet 4) showed that PARP1 associated with multiple DDR proteins (including RAD50, FANCI, FUS, HNRNPA1, HNRNPD, HNRNPA1L2, HSPA4, COIL, and histone H3) (Figure 3B). PARP1 is an important DNA lesion recognizing protein of both the base excision repair (BER) pathway and double-strand break repair (DSBR) pathway. PARP1 modifies chromatin architecture and recruits DDR proteins by mediating the poly-ADP-ribosylation of other DDR players and itself. Our profiling data showed that K418 of PARP1 was mono-methylated. To further confirm this event, we immunoprecipitated PARP1 from the IR-treated cells and analyzed the methylation sites with mass spectrometry. As shown in Supplementary Figure S2A,2B, 10 different mono-methylation sites (K23, K59, K165, R173, K209, K254, K320, K418, K579, and K796) of PARP1 were identified. Among them, the K23 mono-methylation was well enriched in the immunoaffinity-based MS identification in three biological replicates. The level of K23 mono-methylation was approximately 4 times higher in IR-treated cells (*p* < 0.01), and this site has not been reported previously (Figures 3C,D). To examine the role of site-specific methylation of PARP1 in DDR, we generated PARP1 K23A and K418A mutations. While PARP1 K23A mutant showed a compromised response to IR treatment, the K418A mutant had no significant effect (Supplementary Figure S2C). So we focused on the K23 mono-methylation.

### PARP1 K23 Mono-Methylation Functions in the DNA Damage Response

**FIGURE 4:**
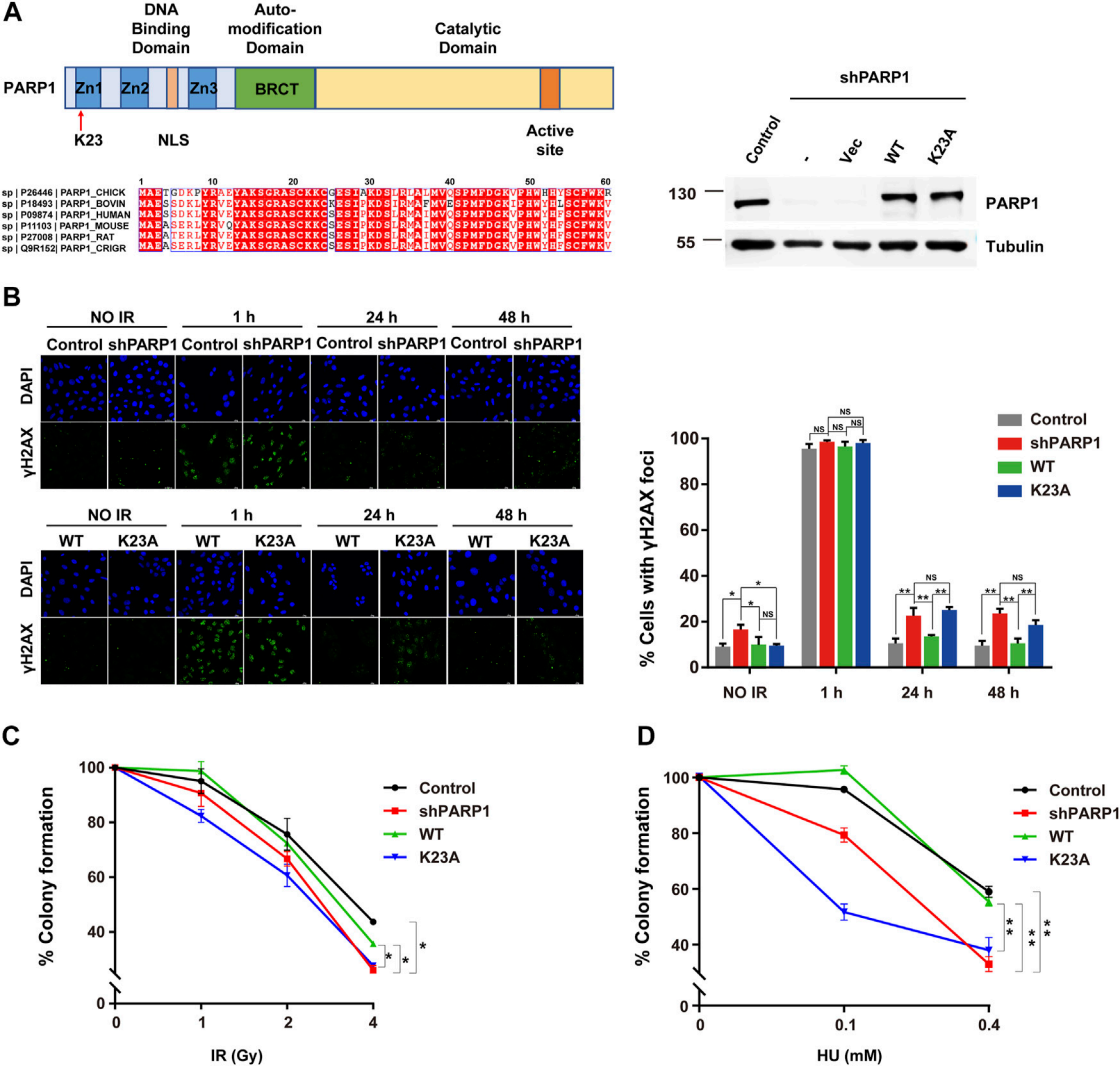
PARP1 K23 mono-methylation functions in the IR-induced DNA damage response. **(A)** Left: Upper panel: scheme of three major domains of the PARP1 protein. Zn1, Zn2, Zn3: Zing fingers; NLS: Nuclear localization signal; BRCT: BRCA1 C-terminus like; K23 site is where the red arrow directs. Lower panel: Alignment of the first 30 amino acids of PARP1 genes from 6 organisms. Right: PARP1 levels in PARP1 knockdown (shPARP1) and WT or K23A reconstituted U2OS cells. **(B)** DNA damage repair shown as the recovery of IR-induced γH2AX foci. Representative γH2AX foci at the indicated times after irradiation (2 Gy) are presented (left panel). Cells with >5 γH2AX foci were counted for quantification. Quantification (right panel) is the average of three independent experiments (100 cells per experiment), presented as the mean ± SD, two-tailed Student’s t test, *p < 0.05. Scale bar: 40 μm. **(C)** Colony formation assay of U2OS stableline cells treated with increasing doses of IR. Data are presented as the mean ± SD of three biological triplicates. The surviving cell percentage was compared with that of the control groupwith two-way ANOVA. *p < 0.05. **(D)** Colony formation assay of U2OS stable line cells treated with increasing doses of HU (right). Data are presented as the mean ± SD of three biological triplicates. The surviving cell percentage was compared with that of the control group with two-way ANOVA. *p < 0.05.

As K23 resides in the first zinc-binding domain (Zn1) of PARP1 (Figure 4A), which is important for DNA-dependent PARP1 activity (Langelier et al., 2011), we speculate that it might participate in the DNA damage sensing and repairing process. To examine whether PARP1 K23 methylation affects the DSB repair, we constructed PARP1 knockdown (shPARP1) and PARP1 WT or K23A reconstituted stable cell lines. The formation and resolution kinetics of γH2AX foci upon IR (2Gy) was detected in these cells to reflect the efficiency of DNA repair. As shown in Figure 4B, depletion of PARP1 resulted in elevated levels of spontaneous γH2AX foci formation and sustained γH2AX foci at 24 h post-IR. Reconstitution with WT PARP1, but not PARP1 K23A mutant, led to resolution of γH2AX foci after 24 h. We further investigated whether K23 methylation affected cellular sensitivity to DNA-damaging agents, including IR and HU. As shown in Figures 4C,D, WT PARP1, but not the K23A mutant, rescued the DNA damage sensitivity conferred by PARP1 deficiency. These results indicate that PARP1 K23 mono-methylation functions in the DNA damage repair process.

### PARP1 K23 Mono-Methylation is Important in the Repair of Stalled Replication Forks

**FIGURE 5:**
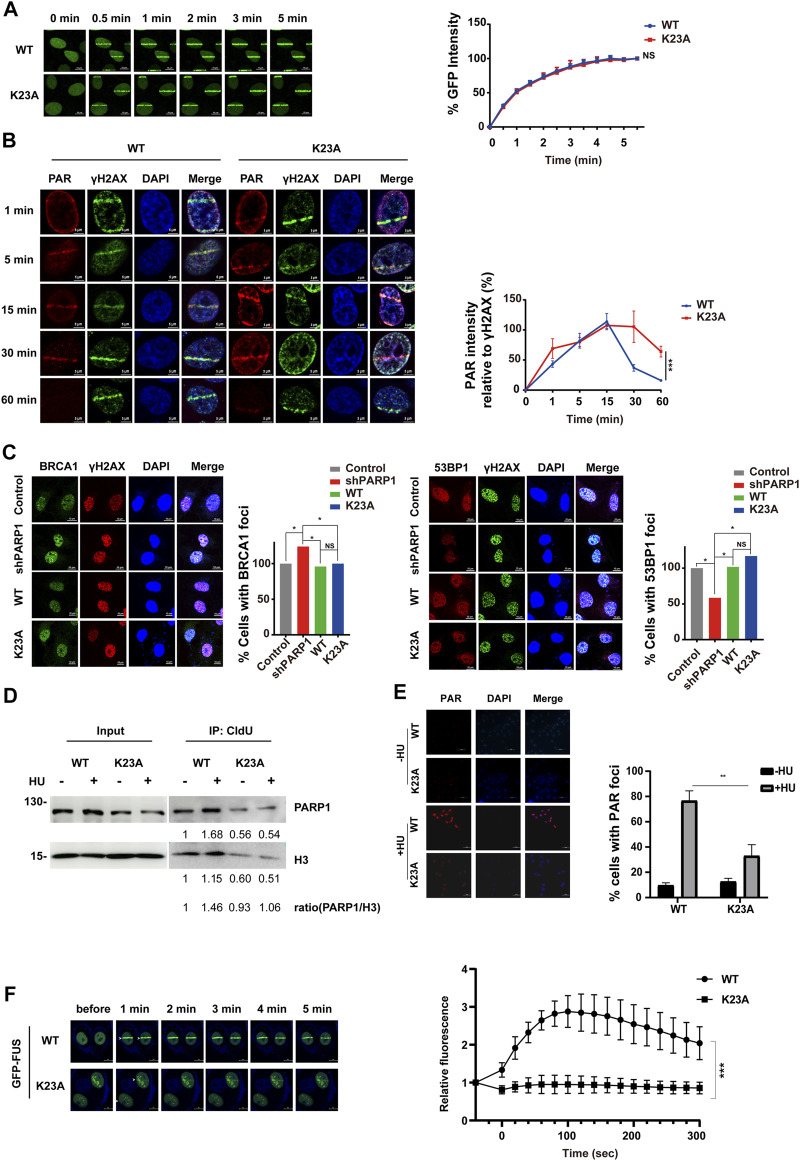
PARP1 K23 mono-methylation is essential for the cell response to replication fork stress. **(A)** Translocation kinetics of PARP1 to DNA damage sites upon UV laser microirradiation. U2OS cells overexpressing EGFP-PARP1 WT or K23A were subjected to laser microirradiation to generate DSBs in a line pattern. The relocation kinetics of EGFP-PARP1 to DSBs were monitored over a time course as indicated. GFP intensities at the laser line were normalized into a numerical value using Nikon NIS-Elements AR software (version 4.40.00). Normalized fluorescence curves from 10 cells were averaged. The error bars represent SD. **(B)** PAR formation at DNA damage sites upon UV laser microirradiation. U2OS cells overexpressing HA-tagged WT or K23A PARP1 were subjected to laser microirradiation to generate DSBs in a line pattern. The kinetics of the PAR polymer at DSBs were monitored over a time course as indicated. PAR immunofluorescence intensities at the laser line were normalized into a numerical value, and PAR laser intensity relative to γH2AX was calculated using Nikon NIS-Elements AR software (version 4.40.00). Normalized fluorescence curves from 6 cells were averaged. The error bars represent SD. **(C)** BRCA1, 53BP1 and γH2AX foci formation in U2OS stable line cells following IR (10 Gy) treatment and a 1 h recovery. Representative images of BRCA1 (left) and 53BP1 (right) foci at sites of laser-induced DNA damage are shown. Scale bar: 10 μm. Quantification of the ratio is the average of three independent experiments (100 cells per experiment), presented as the mean ± SD, two-tailed Student’s t test, *p < 0.05. **(D)** PARP1 associated with stalled replication forks was isolated by CldU coimmunoprecipitation (co-IP) after a 3-h treatment with 2 mM HU. The level of histone H3 was used as a loading control. **(E)** Immunofluorescence staining for PAR in U2OS stable line cells treated with 0.5 mM HU for 24 h. DNA was counterstained with DAPI. Scale bar: 50 μm. **(F)** Translocation kinetics of GFP-FUS to DNA damage sites upon UV laser microirradiation. U2OS cells depleted endogenous PARP1 and reconstituted with WT or K23A were transfected with GFP-FUS plasmid. Cells were then subjected to laser microirradiation to generate DSBs in a line pattern. Representative GFP-FUS localization at sites of laser microirradiation at the indicated times are presented (left panel). Scale bar: 50 μm. The fluorescence values of cells (WT: *n* = 8; K23A: *n* = 10) were normalized to the original signal and plotted as a fluorescence vs. time graph using GraphPad Prism software. The error bars represent SEM. ***p < 0.001.

To investigate the mechanisms by which PARP1 K23 methylation regulates DDR, we first studied whether K23 methylation affects PARP1 itself recruitment to DSB sites. To observe the re-localization kinetics of PARP1 upon UV laser micro-irradiation, we ectopically expressed EGFP-tagged WT and K23A PARP1 in PARP1-depleted cells. A 405 nm laser resulted in similar accumulation kinetics of WT and K23A PARP1 at DSB lesions from 30 s to nearly 5 min post-irradiation (Figure 5A, Supplementary Figure S1A), suggesting that K23 methylation did not compromise PARP1 recruitment to DSB sites. Next, the formation of the PAR polymer at DSB sites was detected, because it reflects the *in situ* enzyme activities of PARP1. As shown in Figure 5B, PAR formation was increased from 1 to 15 min post-laser irradiation in both WT and K23A PARP1-overexpressing cells, while the release of PAR was delayed to a late time point in the K23A mutant cells (30 min–1 h). There are two main pathways responsible for DSB repair, nonhomologous end joining (NHEJ) and homologous recombination (HR). Next, to explore whether K23 methylation influences HR or NHEJ, we examined the accumulation of several DDR factors at DSB sites. We observed compromised 53BP1 foci and increased BRCA1 foci in PARP1 knockdown cells, and both WT and K23A reconstitution rescued the foci phenotypes of the PARP1 knockdown cells (Figure 5C). A minor increase in NHEJ protein 53BP1 foci was shown in K23A-reconstituted cells, suggesting a possible role of K23 methylation in the NHEJ pathway. However, these results were not significant, and the function of PARP1 in DSB repair still needs further verification.

Reports have shown that PARP1 not only plays a role in DSB repair but also is activated at stalled replication forks and contributes to replication stress response (Bryant et al., 2009). We have noticed that the K23 mutant affects the cellular sensitivity to HU-induced replication stress (Figure 4D). To explore how K23 methylation affects the repair of the stalled replication forks, we labelled newly replicated DNA with chlorodeoxyuridine (CldU) after HU stalling and detected the CldU immunoprecipitated PARP1. As shown in Figure 5D, HU treatment led to increased amount of PARP1 at the restarted stalled replication forks in WT PARP1 reconstituted cells. In contrast, K23A mutant compromised the association of PARP1 with the restarted replication forks (Figure 5D), suggesting that K23 methylation influences PARP1 binding with the restarted replication fork structures. To measure PARP1 activation at HU-induced DNA damage sites, we performed a PAR immunofluorescence assay in WT and K23A-reconstituted cells. PAR formation upon HU treatment was obviously decreased in K23A cells than in WT cells, indicating that PARP1 is less activated at sites of stalled and collapsed replication forks in K23A mutant cells (Figure 5E). These results suggested that PARP1 K23 methylation plays a role in the repair of stalled replication forks.

RBPs, such as FUS, CIRBP, SFPQ, have been identified as novel regulators of DDR (Rajesh et al., 2011; Chen et al., 2018; Levone et al., 2021). PARP1 activation is essential for RBPs recruitment to DNA damage sites (Altmeyer et al., 2015). The network analysis revealed that PARP1 associated with multiple RBPs (EWSR1, FUS, ROA1/HNRNPA1, etc.) (Figure 3B). To investigate whether PARP1 K23 methylation affects RBP recruitment, we examined FUS recruitment to DNA damage sites in WT and PARP1 K23A mutant cells. As shown in Figure 5F, FUS recruitment to laser microirradiation-induced DNA damage sites was much weaker in PARP1 K23A mutant cells than WT cells, indicating PARP1 K23 methylation is the promoter step of RBP recruitment.

## Discussion

Protein methylation is an important modification that has been implicated in mRNA splicing, protein translocation, transcriptional control, and DNA repair (Lee et al., 2005; Lake and Bedford, 2007; Erce et al., 2012; Guccione and Richard, 2019). Dysregulation of protein methylation is often associated with cancer, cardiovascular and pulmonary disorders, neuro-degeneration and other diseases (Jarrold and Davies, 2019; Lorton and Shechter, 2019; Urulangodi and Mohanty, 2020). Thus, knowledge of the methylated proteins in a deregulated system might provide promising therapeutic targets. However, it is always challenging to analyze the global methylated proteins in human cells because protein methylation is often sub-stoichiometric. Based on the high hydrophilicity of most tryptic methylated peptides, HILIC (hydrophilic interaction chromatography) enrichment of methylated peptides has been widely used in recent years (Boersema et al., 2008; Uhlmann et al., 2012). Compared with other techniques, HILIC has the advantages of no biases toward methylation forms, fine compatibility with MS, high through-put and low cost. In this work, to investigate the specific protein methylation that exists in the DNA repair process, we established a HILIC-MS strategy for the global analysis of methylated endogenous proteins in response to IR-induced DNA damage. We identified 235 distinct methylation sites in response to IR treatment. The significantly changed methylation covered 49 peptides from 38 proteins. For the first time, our study provides a systematic and comprehensive view of methylated proteins in DNA damage repair networks, which might serve as a valuable resource for future investigation in the DDR process.

Our proteomic analysis detected RBPs as the most abundant proteins in the IR-responding methylome. The RBPs identified in this work include HNRNPD, HNRNPH1, HNRNPH3, HNRNPU, HNRNPK, SPF27/BCAS2, CIRBP, DHX9, SFPQ, FUS, CSTF2, RBMX, THOC4/ALYREF, etc. RBPs are proteins with unique RNA-binding capability that play a major role in RNA processing, mRNA stabilization, mRNA localization, etc. (Lunde et al., 2007; Hentze et al., 2018). In the last decade, quite a few RBPs have been identified as novel regulators of the DSB response (Kai, 2016; Klaric et al., 2021). During DNA repair, they carry out several distinct functions. These functions include, and are not limited to, regulating the recruitment or the activities of DDR factors, remodeling local chromatin, participating in liquid–liquid phase separation around the damage sites. CIRBP, SFPQ and RBMX has been reported to function in the initiation of the DSB response, also in the processes of HR or NHEJ repair (Rajesh et al., 2011; Adamson et al., 2012; Chen et al., 2018). In this study, we identified multiple previously documented methylation sites in SFPQ, CIRBP, RBMX, and in other RBPs. In addition, we also characterized some undocumented methylation sites in RBPs like SPF27/BCAS2, PCBP1, and CPSF2. The functions of specific methylation sites in the DDR remain to be learned and need further exploration. FUS is a multifunctional DNA/RNA-binding protein involved in splicing, translation, and mRNA transport. It was recently reported that FUS-dependent phase separation is important for DNA repair initiation (Levone et al., 2021). As reported, both RGG1 and RGG3 (arginine–glycine–glycine repeat) domains can promote FUS binding to RNA. Here, we found that the levels of R473, R476 and R481 di-methylation in the RGG3 domain were greatly increased upon IR treatment, while the levels of R216 and R218 di-methylation in the RGG1 domain were decreased. Since the RGG3 domain was demonstrated to contribute to FUS phase separation, and both RGG1 and a C-terminal region of RGG3 are hot spots for pathogenic mutations (Corrado et al., 2010; Rademakers et al., 2010; Hofweber et al., 2018), the methylation sites in them need special attention.

In this work, we also observed a PARP1-FUS-centered methylation network in the IR-induced DNA damage response (Figure 3B). Most recently, it was demonstrated that PARP1 activation directs FUS to DNA damage sites, and FUS is required for the recruitment of the DDR factors KU80, NBS1, 53BP1, and SFPQ to DNA damage sites. Previous studies also showed that many other RBPs with intrinsically disordered domains (IDPs) are recruited to DNA damage sites in a PAR-dependent manner (Krietsch et al., 2012; Hong et al., 2013; Chen et al., 2018; Duan et al., 2019; Singatulina et al., 2019). Among the PAR-associated RBPs are FUS, EWSR1, TARF15, multiple hnRNPs, RBM14, etc. These RBPs form liquid compartments around the DNA damage sites. In our study, alterations in the methylation of PARP1, FUS, EWSR1, and many other RBPs in response to IR were identified. We found a novel methylation at K23 in PARP1, which was upregulated due to IR treatment and functioned in DSB and replication fork repair. We demonstrated that K23 methylation especially functions in PAR formation in the DNA repair process (Figure 5E). This finding led to the hypothesis that PARP1 K23 methylation might be the promoter step of RBP recruitment. In a preliminary experiment, we have found that PARP1 K23me is essential for FUS recruitment to microirradiation-induced DNA damage sites (Figure 5F). However, whether PARP1 K23 methylation plays a role in PAR-dependent RBP recruitment has not yet been fully clarified. The detailed function of K23 methylation in the DDR is also complex. In our work, this methylation seemed to carry out more functions in response to replication stresses. We do not know whether this is related to the special structure of the stalled replication fork. More *in vitro* biochemical assays might be performed to answer this question.

The methylations of PARP1 K508, and K528 sites have been previously identified (Kassner et al., 2013; Piao et al., 2014) and have been shown to regulate the activity of PARP1 and assured efficient PAR formation upon oxidative stress. Although such methylations were not observed in our experiments, we noticed that they were obtained through an *in vitro* methyltransferase assay of recombinant PARP1 protein. In our study, these methylations in response to IR-induced DNA damage may have been too sub-stoichiometric to be characterized. Whether the methylation of K508 and K528 functions in DSB repair and replication fork restart needs to be resolved. At the same time, it is also necessary to determine the methyltransferase that targets K23. PARP inhibitors have emerged as promising therapeutics for clinical trials. A previous study showed that the receptor tyrosine kinase c-Met phosphorylates PARP1 at Tyr907. Phosphorylation of PARP1 Tyr907 increases PARP1 enzymatic activity and reduces binding to a PARP inhibitor, thereby rendering cancer cells resistant to PARP inhibition (Du et al., 2016). Combining c-Met and PARP1 inhibitors synergized to suppress the growth of breast cancer cells *in vitro* and lung cancer xenograft tumor models, which highlighted a treatment with a combination of c-Met and PARP inhibitors in patients bearing tumors with high c-Met expression. Here, we also provide some methylations in PARP1 (including K23, K59, K165, R173, K209, K254, K320, K418, K579, and K796). The association of these methylations with DDR and PARPi resistance and the specific methyltransferases targeting these sites also need further detection.

In summary, we have provided a resource for global methylation in response to IR-induced DNA damage. The proteins with changed methylation upon IR treatment include PARP1, FUS, EWSR1, and many other RBPs, which might affect the initial phase separation in the cell response to DNA damage. Nonhistone DDR proteins with novel methylation sites include ADT2, MRNIP, PAPS1, BAG3, SPF27/BCAS2, RAD50, UBP7, FANCI, etc. As previously mentioned, this is the first study to explore the methylome in the DDR using HILIC techniques. However, some limitations of this study should not be ignored. First, we used a single IR treatment to induce DNA damage, which only reflects the context of DSB damage. Second, the HILIC technique adopted in this study could be improved to broaden the detection scope of methylations, such as combined with deglycosylation steps before enrichment (Ma et al., 2017). Therefore, these findings will need further validation in larger datasets.

## Data Availability

The datasets presented in this study can be found in online repositories. The names of the repository/repositories and accession number(s) can be found in the article/Supplementary Material.
