# Supplementary material for: Quantitative Analysis of the Protein Methylome Reveals PARP1 Methylation is involved in DNA Damage Response
Source: Front Mol Biosci. 2022 Jun 29;9:878646. doi: 10.3389/fmolb.2022.878646 (PMC9277342; doi:10.3389/fmolb.2022.878646)
Supplement: Supplementary file 1 [file DataSheet2.docx]

1. The source data of proteomics has been deposited to publicly available datasets, as described in the ‘data availability’ part in the manuscript, as following:

The mass spectrometry proteomics data have been deposited to the ProteomeXchange Consortium via the iProX partner repository (Ma et al., 2019) with the dataset identifier IPX0004099000 (<https://www.iprox.cn//page/project.html?id=IPX0004099000>) / PXD031655 (http://proteomecentral.proteomexchange.org/cgi/GetDataset?ID=PXD031655). ‍

And the working sheets used to analyse these data have been provided in the supplementary materials, as ‘Data sheet 1’ (corresponding to Figure.1 and Figure.2).

1. The code/script file used for figure.2D has been deposited in https://www.jianguoyun.com/p/DSEGukwQ_KamChj-3a4E.

Full scans of the entire original gels displayed in the manuscript, and the original files for the microscopy images have been provided as an .pdf file. This file has been deposited in https://www.jianguoyun.com/p/Db84_J8Q_KamChjyhMcEIAA.

And the cropped images shown in Figure. 4B, 5A, 5B, 5C and 5E have been labeled in red boxes.
