# Supplementary material for: Quantitative Analysis of the Protein Methylome Reveals PARP1 Methylation is involved in DNA Damage Response
Source: Front Mol Biosci. 2022 Jun 29;9:878646. doi: 10.3389/fmolb.2022.878646 (PMC9277342; doi:10.3389/fmolb.2022.878646)
Supplement: Supplementary file 2 [file Image1.PDF]

Supplementary Material

1 Supplementary Data

Datasheet 1. The detailed information of the protein methylome in cellular response to IR-induced DNA damage. Refer to Figure 1 and Figure 2 in the main text for the filtering criteriea.

2 Supplementary Figures

Supplementary Figure 1

Figure S1

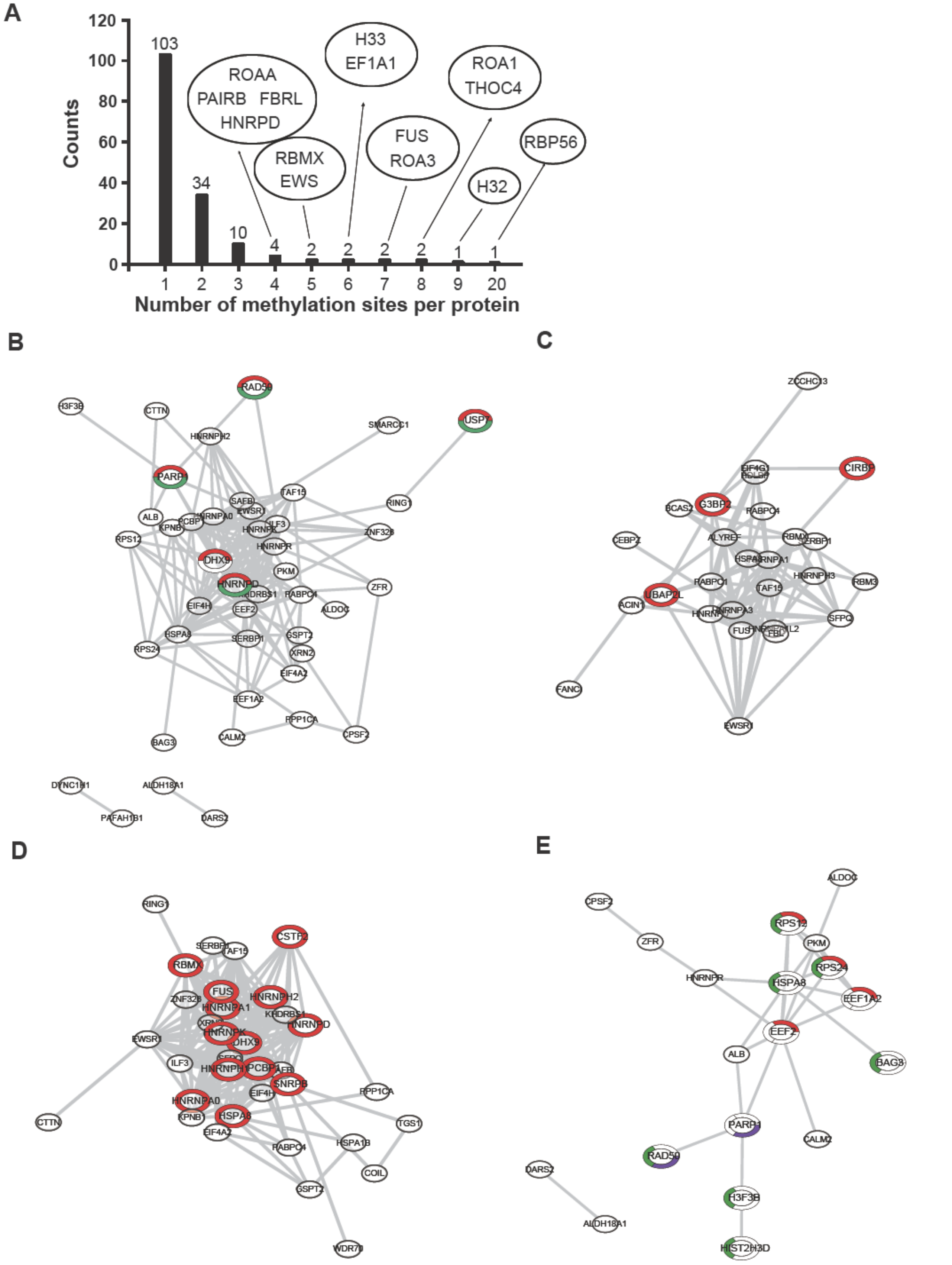
A
Counts
Number of methylation sites per protein
103
34
10
4
2
2
2
2
1
1
ROAA
PAIRB FBRL
HNRPD
RBMX
EWS
H33
EF1A1
FUS
ROA3
ROA1
THOC4
H32
RBP56
B
C
D
E

Supplementary Figure 1. Analysis of identified methylated proteins upon IR treatment.

(A) The distribution of identified methylated proteins in IR-responding methylome with different number of methylation sites. The proteins with more than three methylation sites were shown in oval.

(B) Protein–protein interaction (PPI) network of proteins with increased methylation (log2-fold change>1) upon IR treatment. Methylated proteins derived from the data were analyzed with STRING. The proteins involved in GO:0051054 (Positive regulation of dna metabolic process) and in GO:0032204 (Regulation of telomere maintenance) are highlighted in red and green, respectively.

(C) Protein–protein interaction (PPI) network of proteins with decreased methylation (log2-fold change<-1) upon IR treatment. Methylated proteins derived from the data were analyzed with STRING. The proteins involved in GO:0034063 (Stress granule assembly) are highlighted in red.

(D) Protein–protein interaction (PPI) network of proteins with increased R-methylation (log2-fold change>1) upon IR treatment. Methylated proteins derived from the data were analyzed with STRING. The proteins involved in pathway 'mRNA Splicing - Major Pathway' (HSA-72163) are highlighted in red.

(E) Protein–protein interaction (PPI) network of proteins with increased K-methylation (log2-fold change<-1) upon IR treatment. Methylated proteins derived from the data were analyzed with STRING. The proteins involved in pathways 'Eukaryotic Translation Elongation (HSA-156842)', 'Cellular responses to stress (HSA-2262752)', 'HDR through MMEJ (alt-NHEJ) (HSA-5685939)' are highlighted in red, green, and purple, respectively.

Supplementary Figure 2

Figure S2

A

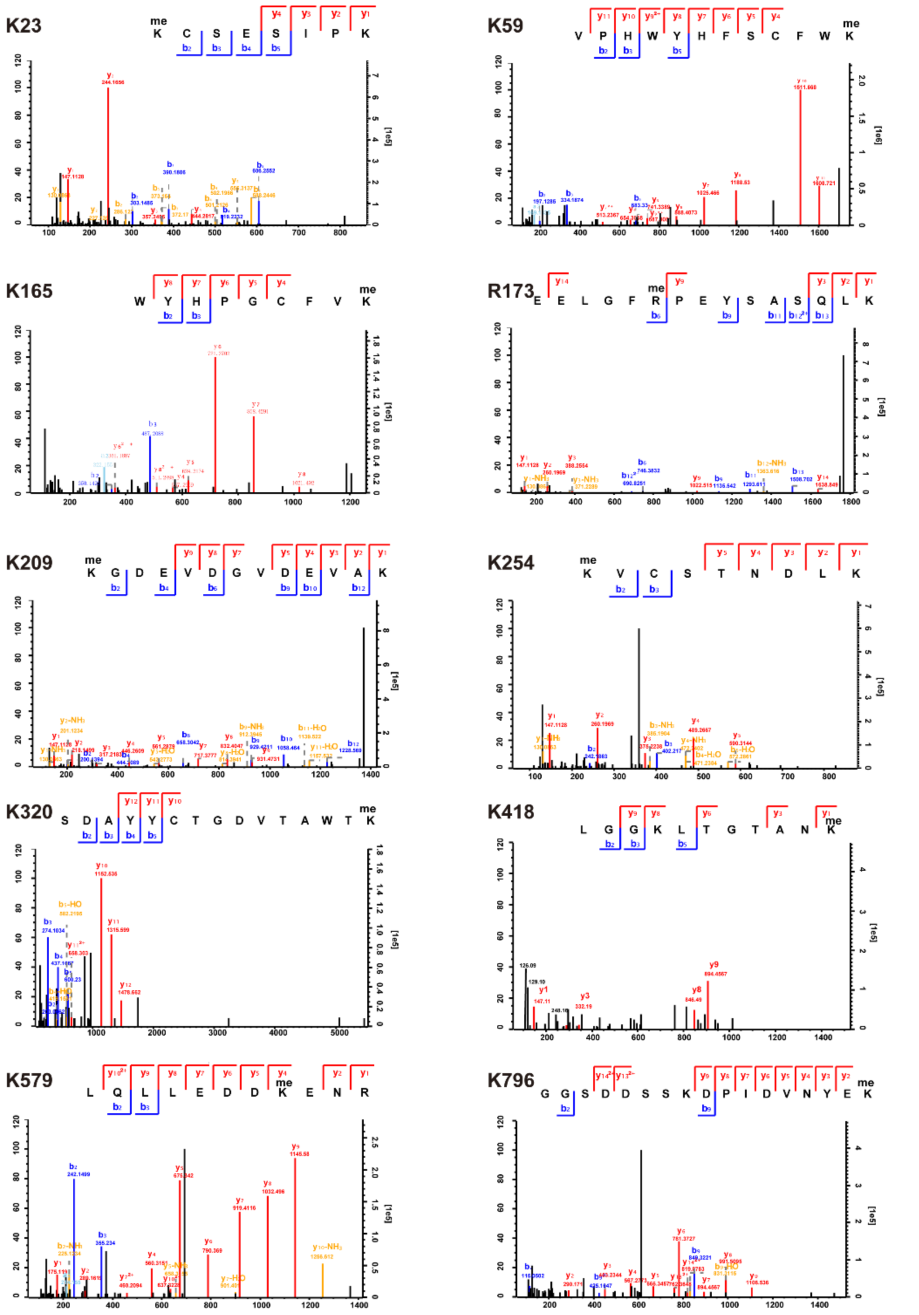

B

methylation sites of PARP1 identified in an MS analysis

site(s)	sequence	abundance in replicates IR	no IR
K23	K*CSESIPK	+/+/+	+/+/+
K59	VPHWYHFSCFWK*	-/-/+	+/-/+
K165	WYHPGCFVK*	+/+/+	+/+/+
R173	EELGFR*PEYSASQLK	-/+/+	+/+/+
K209	K*GDEVDGVDEVAK	-/+/+	+/+/+
K254	K*VCSTNDLK	+/+/+	+/+/+
K320	SDAYYCTGDVTAWTK*	-/-/+	+/-/+
K418	LGGK*LTGTANK	+/+/+	-/+/-
K579	LQLLEDDK*ENR	+/+/+	+/+/-
K796	GGSDDSSKDPIDVNYEK*	+/+/-	+/+/-

C

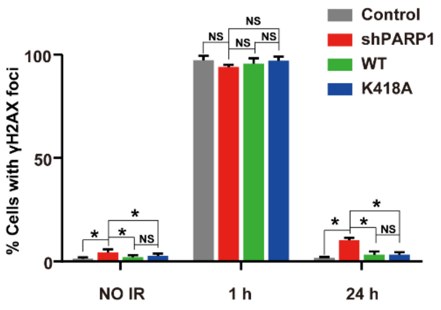

Supplementary Figure 2. Methylation sites of PARP1 identified upon IR treatment.

(A) The MS/MS fragmentation pattern of the identified methylated peptide of PARP1. The identified B ions are shown in blue and Y ions are shown in red.

(B) Summary of the methylation sites of PARP1 identified in an MS analysis. The sites are listed with peptide sequence context, abundance in replicates of IR group and no IR group (+ or - means identified or not identified).

(C) DNA damage repair shown as the recovery of IR-induced γH2AX foci in U2OS cells after irradiation (1 Gy). Cells with >5 γH2AX foci were counted for quantification. Quantification is the average of three independent experiments (100 cells per experiment), presented as the mean ± SD, two-tailed Student's t test, *$P < 0.05$.
